# New Classification for the Reporting of Complications in Retinal Detachment Surgical Trials

**DOI:** 10.1001/jamaophthalmol.2021.1078

**Published:** 2021-06-24

**Authors:** Zheng Yang Xu, Augusto Azuara-Blanco, Kazuaki Kadonosono, Timothy Murray, Sundaram Natarajan, Samantha Sii, William Smiddy, David H. Steel, Thomas J. Wolfensberger, Noemi Lois

**Affiliations:** 1 Centre for Public Health, Queen’s University Belfast, Belfast, Northern Ireland; 2 Department of Ophthalmology, Yokohama City University Medical Center, Yokohama, Japan; 3 Bascom Palmer Eye Institute, University of Miami Miller School of Medicine, Miami, Florida; 4 Aditya Jyot Eye Hospital, Mumbai, India; 5 Department of Ophthalmology, Lincoln County Hospital, Lincoln, England; 6 Bascom Palmer Eye Institute, Miami, Florida; 7 Sunderland Eye Infirmary, and Institute of Genetic Medicine, University of Newcastle Upon Tyne, Newcastle Upon Tyne, England; 8 Jules Gonin Eye Hospital, University of Lausanne, Lausanne, Switzerland

## Abstract

**Importance:**

Quantifying severity of complications in rhegmatogenous retinal detachment (RRD) surgical trials is needed. A consensus classification system will help surgeons to more effectively compare harms of different surgical techniques.

**Objective:**

To develop a new consensus-based classification to quantify severity of complications of RRD surgery.

**Design, Setting, and Participants:**

A comprehensive list of complications was developed followed by a Delphi consensus survey of international vitreoretinal surgeons. The survey was conducted in 17 countries in mainland Europe, the United Kingdom, the United States, Asia, South Africa, and Australia. Seventy vitreoretinal surgeons were invited to take part in the Delphi survey; 45 agreed to participate. Participants were selected through boards/members lists of retinal societies. Data were analyzed between April 2019 and August 2019.

**Main Outcomes and Measures:**

Consensus-derived classification of complications of RRD surgery, according to their severity.

**Results:**

Forty-three of 45 vitreoretinal surgeons who agreed to participate in the Delphi survey completed round 1 (96%); all but 1 (98%) completed round 2. Consensus was reached for 96% of the 84 complications assessed. Examples of complications classified as least severe (graded 1) included subconjunctival hemorrhage and chemosis while those classified as most severe included endophthalmitis and sympathetic ophthalmia (graded 9) and phthisis (graded 10).

**Conclusions and Relevance:**

We propose a new classification for quantifying severity of surgical complications based on an international consensus of vitreoretinal surgeons to quantify harm and improve the reporting of complications of RRD surgery.

## Key Points

**Question:**

Can consensus classification for complication severity be developed to be used in clinical trials of rhegmatogenous retinal detachment (RRD) surgery?

**Findings:**

In this study, following the development of a comprehensive list of complications of RRD surgery, a Delphi survey among retinal surgeons was undertaken (96% response rate). Consensus was achieved in grading severity of complications of RRD surgery.

**Meaning:**

The proposed severity classification for complications of RRD surgery may facilitate quantification and comparison of harm of different RRD surgical interventions.

## Teaser

This study analyzes a survey of international vitreoretinal surgeons to develop a new consensus-based classification to quantify severity of complications of rhegmatogenous retinal detachment surgery.

## Introduction

Rhegmatogenous retinal detachment (RRD) is the most common retinal emergency, carrying an annual incidence of 6.3 to 17.9 per 100 000 population, and is one of the most common indications for vitreoretinal intervention.^1,2^ Surgical techniques and maneuvers used to repair RRD have evolved over decades such that high rates of retinal reattachment are currently achieved with pars plana vitrectomy (PPV), scleral buckle (SB), pneumatic retinopexy (PR), or combinations thereof.^3,4,5^

Randomized clinical trials (RCTs) provide the best evidence to guide surgeons in the selection of interventions to optimize success for patients.^6,7,8,9^ When comparing surgical procedures, it is important that these interventions are evaluated comprehensively with regard to their potential harms as well as their effectiveness.^10^ There is evidence that the reporting of complications in clinical trials across many surgical and medical specialties lacks sufficient quality and consistency.^11,12,13,14,15^ For example, although RCTs commonly report complication frequency, data on severity of complications are often lacking.^16,17,18^ While classification systems for severity of surgical complications have been proposed and used in fields such as general surgery, neurosurgery, and urology,^19,20,21,22,23^ to our knowledge, these have not been incorporated into ophthalmic RCTs. In response to this need, in 2018, Sii et al^24^ published a grading system for complications of glaucoma surgery.

With this in mind, the purpose of the study was to reach consensus among an international group of vitreoretinal surgeons on severity scores for complications of RRD surgery and to generate a classification system for quantifying and reporting severity of complications of retinal detachment surgery (CORDS).

## Methods

Three authors (Z.Y.X., N.L., and A.A.B.) generated first a preliminary list of complications of RRD surgery using a textbook on complications of vitreoretinal surgery.^25^ This pilot list was then distributed to a small group of vitreoretinal surgeons (authors K.K., T.M., S.N., D.S., W.S., and T.W.) who provided feedback and guided the establishment of a final list. This was developed into a survey to determine the severity of each of the complications in the list; the survey was prepared for distribution using the software platform Enalyzer PRO (Enalyzer).^26^ A pilot phase was run among 7 vitreoretinal surgeons (K.K., N.L., T.M., S.N., D.S., W.S., and T.W.) to refine the survey questions and test the user interface.

A consensus on the grading of complications of RRD surgery was pursued using the Delphi consensus method. The Delphi method comprises 2 or more rounds of anonymous surveys where responses are summated after each round and presented to the participants in the subsequent round. At each stage, respondents may choose to use the summary information to modify their responses from the previous round or opt to maintain their previous answers. The range of responses for each survey item tends to decrease with each cycle such that the group converges toward a consensus response.^27^

Members of several retinal societies including Macula Society, the Club Jules Gonin, and the British and Eire Association of Vitreoretinal Surgeons (BEAVRS) were invited to take part in the survey to gather an international and representative group of vitreoretinal surgeons (n = 70 from 17 countries). Members were selected from societies’ member lists to ensure there would be international representation from all continents. Institutional ethical review approval was obtained (Queen’s University Belfast Faculty research ethics committee ). All survey participants provided written informed consent; none received a stipend for their participation.

Survey participants were presented with complications of RRD surgery divided into general, common complications, and complications specific to SB, PPV, and PR. Each category was subdivided into intraoperative and postoperative complications (eTable 1 in the Supplement). Participants were asked to rank each complication from 1 to 10, with 1 representing “no harm to patient or vision” and 10 “worst possible harm to the patient or vision (eg, permanent total loss of vision or painful eye).” Unlike many other surgical specialties, mortality is a rare complication of ophthalmic surgery and was excluded along with complications from anesthesia. Aside from anchor statements for the least and worst grades of the scale (1 and 10), no additional guidance was provided. In addition, space was provided to allow vitreoretinal surgeons to justify their responses or provide feedback on the questions. The questions contained in the round 1 of the survey can be accessed on https://surveys.enalyzer.com?pid=rab5r4tg.

Round 1 responses were extracted from the software and analyzed in Excel (Microsoft), and the median severity grade and interquartile range (IQR) for each complication was calculated. In round 2, participants were presented with both median scores from the previous round and their own previous scores. Vitreoretinal surgeons then assigned their scores to the complications presented in the second round. Data were then reanalyzed and the survey cycle was to be repeated until consensus on more than 90% of the complications had been achieved. For the purposes of the survey, an IQR of 2 or less was required for a grade on the severity of the complication to be judged to have reached consensus. Items that reached consensus in the first round of the Delphi were removed from the second round of questioning. Comments made in round 1 were taken into consideration for the preparation of the second round, resulting in minor modifications to the survey items. The specific changes that were made allowed us to capture how severity scores differed depending on the size of suprachoroidal and subretinal hemorrhage, whether the macula was involved, whether visual field loss affects driving, and whether fish-egg gas bubble formation affects the view of the retina. The round 2 questions can be accessed in https://surveys.enalyzer.com?pid=t7h4gaqb.

## Results

**Figure 1.**
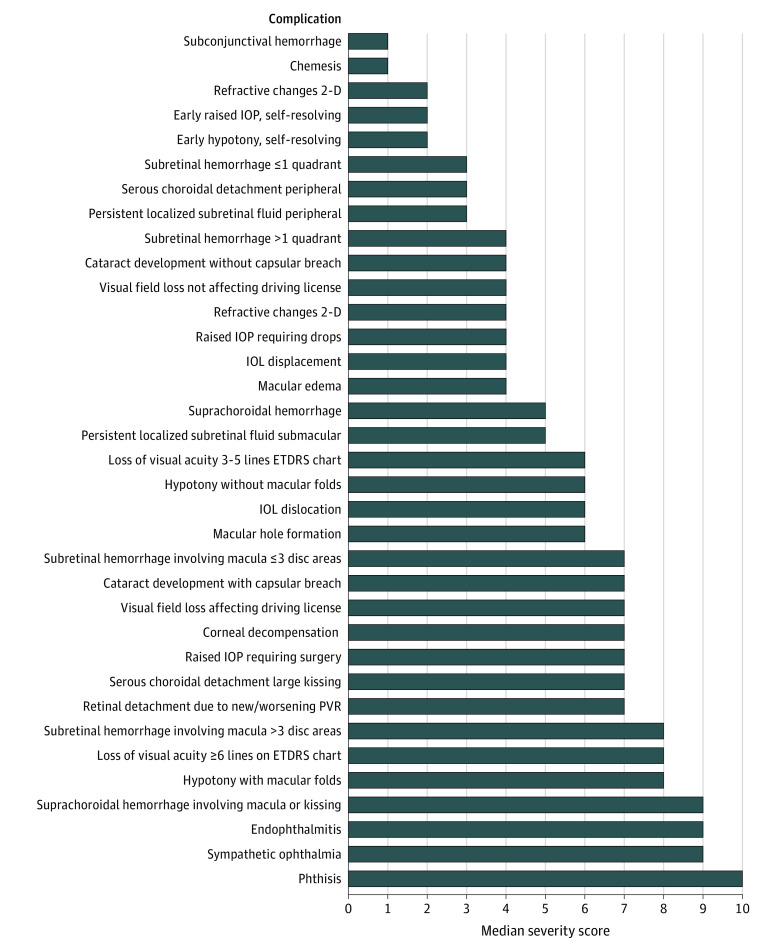
Complications of Surgery for the Repair of Rhegmatogenous Retinal Detachment, Ranked by Median Severity Score General complications of surgery (pars plana vitrectomy, scleral buckling, and pneumatic retinopexy) for the repair of rhegmatogenous retinal detachment, ranked by median severity score. 2-D indicates 2-dimensional; IOP, intraocular pressure; IOL intraocular lens; ETDRS, Early Treatment Diabetic Retinopathy Study; PVR, proliferative vitreoretinopathy.

**Figure 2.**
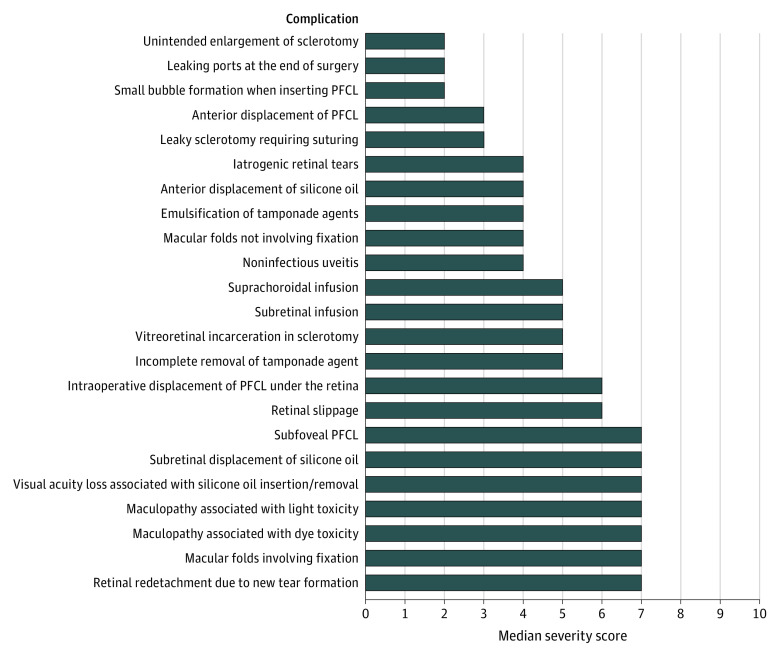
Complications of Pars Plana Vitrectomy PFCL indicates perfluorocarbon liquid.

**Figure 3.**
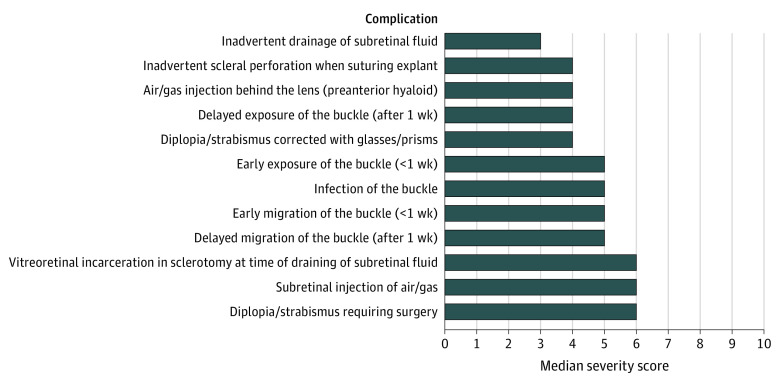
Complications of Scleral Buckling

**Figure 4.**
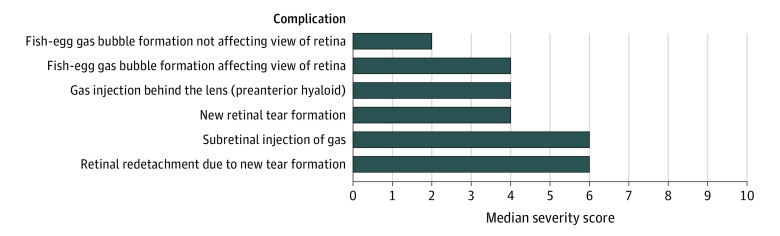
Complications of Pneumatic Retinopexy

Forty-five of the 70 vitreoretinal surgeons approached (64%) replied to the initial contact and agreed to participate in the Delphi survey; 43 (96%) of these completed round 1 in full. Participants in the first round of the Delphi were based in mainland Europe (n = 14; 33%), United Kingdom (n = 10; 23%), United States (n = 10; 23%), Asia (n = 5; 12%), South Africa (n = 3; 7%), and Australia (n = 1; 2%). A consensus was reached for 32 of 78 complications (41%) in round 1 (IQR≤2); consensus items were removed from the round 2 question list. The remaining list of nonconsensus items comprised 46 items, which, taking into account feedback from round 1, was expanded to 55 items. The completion rate for round 2 of the survey was 98%; and 52 of 55 items (95%) achieved a consensus severity grading. Combining the results from round 1 and 2, the final list comprises 87 complications, of which 84 (97%) reached consensus. The 3 complications for which consensus was not reached were “suprachoroidal hemorrhage, not kissing and not involving the macula”; “subretinal infusion” in the context of PPV; and “early migration of the scleral buckle”; these achieved an IQR of 2.75. Full round 1 and 2 results can be accessed online (eTable 2 in the Supplement). The final list of complications with their assigned median severity gradings is shown in the Table. Figure 1, Figure 2, Figure 3, and Figure 4 present all complications ranked based on their scores; eTable 3 in the Supplement presents complications ranked and classified as mild, moderate, or severe.

**Table.** Median Scores for General and Specific Complications by Surgical Procedure Using the Complications of Retinal Detachment Surgery (CORDS) Severity Classification

Complication	Median
General intraoperative	
Subconjunctival hemorrhage	1
Chemosis	1
Subretinal hemorrhage not involving macula, ≤1 quadrant	3
Subretinal hemorrhage not involving macula, >1 quadrant	4
Suprachoroidal hemorrhage not involving macula and no kissing^a^	5
Subretinal hemorrhage involving macula, ≤3 disc areas	7
Subretinal hemorrhage involving macula, >3 disc areas	8
Suprachoroidal hemorrhage involving macula or kissing	9
General postoperative	
Subconjunctival hemorrhage	1
Chemosis	1
Refractive changes: <2-dimensional	2
Early raised IOP, self-resolving	2
Early hypotony, self-resolving	2
Serous choroidal detachment: peripheral	3
Persistent localized subretinal fluid: peripheral, nonprogressive	3
Visual field loss not related to retinal detachment but attributable to surgical procedure: not affecting driving license	4
Refractive changes: ≥2-dimensional	4
Persistently raised IOP manageable with drops	4
IOL displacement	4
Macular edema	4
Suprachoroidal hemorrhage: not involving macula and no kissing	5
Persistent localized subretinal fluid: submacular	5
Loss of visual acuity attributable to surgical procedure: moderate (3-5 lines ETDRS chart)	6
Persistent hypotony (IOP <5 mm Hg) without macular folds	6
IOL dislocation	6
Macular hole formation	6
Visual field loss not related to retinal detachment but attributable to surgical procedure: affecting driving license	7
Corneal decompensation/severe corneal edema	7
Persistently raised IOP requiring surgery	7
Serous choroidal detachment: large, kissing	7
Retinal redetachment owing to new or worsening PVR	7
Loss of visual acuity attributable to surgical procedure: severe (≥6 lines on ETDRS chart)	8
Persistent hypotony with macular folds	8
Suprachoroidal hemorrhage: involving macula or kissing	9
Endophthalmitis	9
Sympathetic ophthalmia	9
Phthisis	10
PPV intraoperative	
Unintended enlargement of sclerotomy	2
Leaking ports at the end of surgery requiring suturing	2
Small bubble formation when inserting PFCL	2
Cataract development intraoperatively (owing to lens touch): without capsular breach	4
Iatrogenic retinal tears	4
Suprachoroidal infusion	5
Subretinal infusion^a^	5
Cataract development intraoperatively (owing to lens touch): with capsular breach	5
Vitreoretinal incarceration in sclerotomy	5
Intraoperative displacement of PFCL under the retina	6
PPV postoperative	
Anterior displacement of tamponade agent: PFCL	3
Leaky sclerotomy requiring suturing	3
Anterior displacement of tamponade agent: silicone oil	4
Emulsification of tamponade agents	4
Macular folds: not involving fixation	4
Noninfectious uveitis	4
Incomplete removal of tamponade agent	5
Retinal slippage	6
Subfoveal PFCL	7
Subretinal displacement of silicone oil and heavy silicone oils	7
Unexplained visual acuity loss associated with insertion/removal of silicone oil	7
Maculopathy related to light toxicity	7
Maculopathy related to dye toxicity	7
Macular folds: involving fixation	7
Retinal redetachment owing to new tear formation	7
SB intraoperative	
Inadvertent drainage of subretinal fluid	3
Inadvertent scleral perforation when suturing explant	4
Air/gas injection behind the lens (preanterior hyaloid)	4
Cataract development intraoperatively (due to lens touch, eg, at time of injection of air/gas): without capsular breach	4
Vitreoretinal incarceration in sclerotomy at time of draining of subretinal fluid	6
Subretinal injection of air/gas	6
Cataract development intraoperatively: with capsular breach	7
SB postoperative	
Delayed exposure of the buckle (after 1 wk)	4
Diplopia/strabismus: corrected with glasses/prisms	4
Early exposure of the buckle (<1 wk)	5
Infection of the buckle	5
Early migration of the buckle (<1 wk)^a^	5
Delayed migration of the buckle (after 1 wk)	5
Diplopia/strabismus: requiring surgery	6
PR intraoperative	
Fish-egg gas bubble formation not affecting view of retina	2
Fish-egg gas bubble formation affecting view of retina	4
Gas injection behind the lens (preanterior hyaloid)	4
Cataract development intraoperatively (owing to lens touch at the time of gas injection): without capsular breach	4
Subretinal injection of gas	6
Cataract development intraoperatively: with capsular breach	7
PR postoperative	
New retinal tear formation	4
Retinal redetachment owing to new tear formation	6

Abbreviations: D, diopters; ETDRS, Early Treatment Diabetic Retinopathy Study; IOP, intraocular pressure; PFCL, perfluorocarbon liquid; PPV, pars plana vitrectomy; PR, pneumatic retinopexy; SB, scleral buckle.

^a^ Interquartile range = 2.75 (ie, did not reach consensus).

## Discussion

Using the Delphi method of consensus, we developed a comprehensive classification to quantify severity of complications of RRD surgery. Capturing data on severity of complications is important for comparing risks-benefits of new or emerging interventions. For example, a new surgical procedure found to be noninferior with regard to efficacy and to have equal frequency of complications to the standard surgery in an RCT may be considered a potential option for the management of a particular condition. However, this may not be the case if in the same scenario, complications observed following the new approach were identified to be more severe than those occurring following standard surgery.^28^

Several classifications of severity of surgical complications have been developed in other specialties. An example is the Clavien-Dindo classification, a 5-grade system for complications of general surgery that has been widely adopted in surgical trials since 2004.^29,30,31,32^ Others commonly used include the Accordion and Memorial Sloan Kettering Cancer Center classifications.^33,34^ There are few comparable grading systems adapted to the field of ophthalmology. A systematic review conducted by our group to inform this project found that RCTs on RRD surgery do not report well on harms associated with surgery nor did they quantify severity of complications. Recently, a grading system for complications of glaucoma surgery has been published and has shown potential for implementation in future trials in this area.^24^ These classifications should provide an additional tool in facilitating a more complete understanding, analysis, and interpretation of results in ophthalmic surgical RCTs.

We adhered as far as possible to the original “Clavien” definition of a surgical complication as “a deviation from the normal postoperative course, which cannot otherwise be classed as either a sequela (an ‘after effect’ that is inherent to the procedure) or a failure to cure” in developing our complication list.^29^ There are other important considerations that extend beyond the scope of the current study. Subjective patient experiences, for example, the reporting of pain, or patient’s opinion of complications severity could be explored in the future. Similarly, complications related to anesthesia were not investigated because they have dedicated representation in the literature.^35,36,37,38^

For patient and surgeon, ultimately a most important metric of complication severity is the visual outcome. Measuring relative effects of all possible surgical complications on vision would be daunting. Expert focus groups have been used in such circumstances in an attempt to resolve differences in opinion among surgeons.^39,40,41^ This is important because individual surgeon experiences may vary significantly, and the perceived severity of a surgical complication is likely to depend on the context in which it occurs. For instance, a complication may be perceived as being more severe if it occurs in an only eye or in an eye with significant visual potential. Human factors, such as prior experience of the operating surgeon in managing a particular complication, may also be relevant in this regard. These challenges make the development of a classification an excellent substrate for the Delphi method. Similar to an expert focus group, a Delphi survey assimilates information from a range of experts within a field. However, crucially, it carries the additional advantages of anonymity and equal weighting of all contributors, removing the potential influence of “strong personalities” and allowing controlled and structured feedback, guiding the group more systematically toward a convergence of opinion.^27^ The strengths of the method are supported by the high degree of consensus achieved in our study (97% of all items) among surgeons with a range of backgrounds and clinical experience.

For the classification to be used widely by vitreoretinal surgeons, it is essential for it to be made accessible in a user-friendly manner to all. For this reason, we are in the process of creating a free app, expected to be available in autumn of 2021, through smartphones and computers, which will contain all complications with the corresponding severity scores. Thus, vitreoretinal surgeons will be able to incorporate the list of expected complications and their severity scoring to the protocol of clinical trials so that surgical procedures could be compared not only with regard to efficacy but also harms. It will be possible not only to report homogeneously intraoperative and postoperative complications in future RTCs, based on the comprehensive list proposed, but also to quantify harm by providing frequency of complications based on their scores or severity (mild, moderate, or severe) and potentially use a harm score (eg, score of each severe complication multiplied by the number of cases with the complication). The severity classification could be used also for the purpose of clinical auditing of surgical results and surgical registries.

### Strengths and Limitations

This study has strengths and limitations. We used the Delphi approach, which is a robust and well-established method of generating consensus from a group of experts regarding a highly specialized topic.^27^ The reproducibility of the Delphi method among panels of experts in a particular area of interest has been confirmed in several studies.^42,43^ The sample size of our study is adequate as the survey targeted experts who have similar training and general understanding in the field of interest.^27^ We were unable to reach consensus in all complications. In Delphi studies, it is important to determine an appropriate a priori definition of consensus as well as an appropriate end point of the study.^44,45^ In quantitative Delphi studies, according to 1 systematic review, a commonly reported definition of consensus has been an IQR of 3 or less on a 9-point scale.^46^ Thus, under these criteria, our study would have achieved 100% consensus. However, we opted to use instead for a more stringent definition (IQR ≤2 on a 10-point scale), and we were able to reach consensus in 97% of the complications in 2 rounds of surveying. Future ways of validating CORDS may involve testing the classification against the patient’s perspective using patient-reported outcome measures.^47^

## Conclusions

We believe having a comprehensive list of complications of RRD surgery with quantitative severity scores for each will improve the quality of the design and reporting of surgical RCTs, conforming with CONSORT recommendations for harms reporting, and ultimately will allow vitreoretinal surgeons to better select surgical procedures for their patients, based not only on their comparative effectiveness but also on their harms. The upcoming free app will facilitate its implementation.

## Notes

Supplement. **eTable 1.** Preliminary list of complications of retinal detachment surgery used to develop the Delphi survey **eTable 2.** Delphi Survey – Full round 1 and 2 results **eTable 3.** Complications ranked by severity scores and classified as mild (median scores 1-4), moderate (median scores 5-7) and severe (median scores 8-10)
