# Supplementary material for: New Classification for the Reporting of Complications in Retinal Detachment Surgical Trials
Source: JAMA Ophthalmol. 2021 Jun 24;139(8):857–64. doi: 10.1001/jamaophthalmol.2021.1078 (PMC8227451; doi:10.1001/jamaophthalmol.2021.1078)
Supplement: Supplement. — eTable 1. Preliminary list of complications of retinal detachment surgery used to develop the Delphi survey eTable 2. Delphi Survey – Full round 1 and 2 results eTable 3. Complications ranked by severity scores and classified as mild (median scores 1-4), moderate (median scores 5-7) and severe (median scores 8-10) [file jamaophthalmol-e211078-s001.pdf]

# Supplemental Online Content

Xu ZY, Azuara-Blanco A, Kadonosono K, et al; the CORDS Study Group. New classification for the reporting of complications in retinal detachment surgical trials. *JAMA Ophthalmol*. Published online June 24, 2021. doi:10.1001/jamaophthalmol.2021.1078

**eTable 1.** Preliminary list of complications of retinal detachment surgery used to develop the Delphi survey

**eTable 2.** Delphi Survey – Full round 1 and 2 results

**eTable 3.** Complications ranked by severity scores and classified as mild (median scores 1-4), moderate (median scores 5-7) and severe (median scores 8-10)

This supplemental material has been provided by the authors to give readers additional information about their work.

© 2021 Xu ZY et al. *JAMA Ophthalmology*.

## eTable 1: Preliminary list of complications of retinal detachment surgery used to develop the Delphi survey

| **1. General complications** | **Score (1-10)** |
|---|---|
| **1a. Intra-operative** | |
| Intra-operative subconjunctival haemorrhage | |
| Intra-operative chemosis | |
| Intra-ocular bleeding: | |
| - Subretinal haemorrhage not involving macula | |
| - Subretinal haemorrhage involving macula | |
| - Suprachoroidal haemorrhage not involving macula | |
| - Suprachoroidal haemorrhage involving macula | |
| **1b. Post-operative** | |
| Post-operative subconjunctival haemorrhage | |
| Post-operative chemosis | |
| Loss of visual acuity attributable to surgical procedure: | |
| - Moderate (loss of 3-5 lines on ETDRS chart) | |
| - Severe (loss of ≥6 lines on ETDRS chart) | |
| Visual field loss not related to retinal detachment but attributable to the surgical procedure: | |
| - Mild (<1 quadrant, not involving fixation) | |
| - Severe (≥ 1 quadrant, or involving fixation) | |
| Refractive changes | |
| - <2D | |
| - ≥2D | |
| Corneal decompensation / severe corneal oedema | |
| IOP-related | |
| - Early high IOP self-resolving | |
| - Persistent high IOP manageable with drops | |
| - Persistent high IOP requiring surgery | |
| - Persistent hypotony (IOP < 5mmHg) without macular folds | |
| - Persistent hypotony with macular folds | |
| Phthisis | |
| IOL displacement | |
| IOL dislocation | |
| Serous choroidal detachment | |
| - Peripheral | |
| - Large, kissing | |
| Post-op suprachoroidal haemorrhage | |
| - Peripheral | |
| - Involving macula | |
| Symptomatic epimacular membrane | |
| Post-op macular oedema | |
| Post-op macular hole formation | |
| Persistent localised subretinal fluid | |
| - Peripheral, non-progressive | |
| - Submacular | |
| Retinal re-detachment due to PVR | |
| Endophthalmitis | |
| Sympathetic ophthalmia | |
| **2. Complications relating to pars-plana vitrectomy** | |
| **2a. Intra-operative** | |
| Unintended enlargement of sclerotomy | |
| Suprachoroidal / subretinal infusion | |

© 2021 Xu ZY et al. *JAMA Ophthalmology*.

| | |
|---|---|
| Leaking ports at the end of surgery requiring suturing | |
| Cataract development intra-operatively (due to lens touch) | |
| - Without capsular breach | |
| - With capsular breach | |
| Related to tamponade agents: | |
| - Intra-operative displacement of perfluorocarbon liquid (PFCL) under the retina | |
| - Small bubble formation when inserting PFCL | |
| - ‘Sticky’ silicone oil at the time of removal | |
| Iatrogenic retinal tears | |
| Vitreo-retinal incarceration in sclerotomy | |
| **2b. Post-operative** | |
| Anterior displacement of tamponade agent | |
| - PFCL | |
| - Silicone Oil | |
| Subfoveal PFCL | |
| Subretinal displacement of silicone oil and heavy silicone oils | |
| Incomplete removal of tamponade agent | |
| Unexplained visual acuity loss associated with insertion / removal of silicone oil | |
| Emulsification of tamponade agents | |
| Non-infectious uveitis | |
| Leaky sclerotomy requiring suturing | |
| Post-op maculopathy related to light toxicity | |
| Post-op maculopathy related to dye toxicity | |
| Macular folds | |
| - Involving fixation | |
| - Not involving fixation | |
| Retinal slippage | |
| Retinal re-detachment due to new tear formation | |
| **3. Complications relating to scleral buckling** | |
| **3a. Intra-operative** | |
| Vitreo-retinal incarceration in sclerotomy at the time of draining of subretinal fluid | |
| Inadvertent scleral perforation when suturing explant | |
| Inadvertent drainage of subretinal fluid | |
| Air/ gas injection behind the lens | |
| Subretinal injection of air / gas | |
| Cataract development intra-operatively (due to lens touch, e.g. at time of injection of air/gas) | |
| - Without capsular breach | |
| - With capsular breach | |
| **3b. Post-operative** | |
| Complications relating to the buckle | |
| - Exposure | |
| - Migration | |
| - Infection | |
| Diplopia/ strabismus | |
| - Corrected with glasses / prisms | |
| - Requiring surgery | |
| **4. Complications relating to pneumatic retinopexy** | |
| **4a. Intra-operative** | |
| ‘Fish-egg’ gas bubble formation | |
| Gas injection behind the lens | |
| Sub-retinal injection of gas | |
| Cataract development intra-operatively (due to lens touch, at the time of injection of gas) | |
| - Without capsular breach | |
| - With capsular breach | |
| **4b. Post-operative** | |
| New retinal break formation | |
| Retinal re-detachment due to new tear formation | |

© 2021 Xu ZY et al. *JAMA Ophthalmology*.

**eTable 2a: Delphi Round 1 Results: Median and IQR scores for complications by technique: pars-plana vitrectomy (PPV), scleral buckle (SB), pneumatic retinopexy (PR), and whether intra-operative (IO) or post-operative (PO)**

| Category | Complication | Median | IQR |
|---|---|---|---|
| General IO | Subconjunctival haemorrhage | 1 | 0 |
| | Chemosis | 1 | 1 |
| | Subretinal haemorrhage not involving macula | 3 | 1 |
| | Subretinal haemorrhage involving macula | 7 | 1.5 |
| | Suprachoroidal haemorrhage not involving macula | 5 | 3 |
| | Suprachoroidal haemorrhage involving macula | 9 | 2.5 |
| General PO | Subconjunctival haemorrhage | 1 | 0 |
| | Chemosis | 1 | 0 |
| | Loss of visual acuity attributable to surgical procedure: Moderate (3-5 lines ETDRS chart) | 6 | 2 |
| | Loss of visual acuity attributable to surgical procedure: Severe (≥ 6 lines on ETDRS chart) | 8 | 2 |
| | Visual field loss not related to retinal detachment but attributable to surgical procedure: Mild (< 1 quadrant, not involving fixation) | 5 | 3 |
| | Visual field loss not related to retinal detachment but attributable to surgical procedure: Severe (≥ 1 quadrant or involving fixation) | 7 | 1 |
| | Refractive changes: < 2D | 2 | 1 |
| | Refractive changes: ≥ 2D | 4 | 2 |
| | Corneal decompensation / severe corneal oedema | 7 | 2 |
| | Early raised IOP, self-resolving | 2 | 1 |
| | Persistently raised IOP manageable with drops | 4 | 2 |
| | Persistently raised IOP requiring surgery | 7 | 2 |
| | Persistent hypotony (IOP < 5mmHg) without macular folds | 6 | 2 |
| | Persistent hypotony with macular folds | 8 | 2 |
| | Phthisis | 10 | 0 |
| | IOL displacement | 4 | 1.5 |
| | IOL dislocation | 6 | 3 |
| | Serous choroidal detachment: peripheral | 3 | 1 |
| | Serous choroidal detachment: large, kissing | 7 | 2 |
| | Suprachoroidal haemorrhage: peripheral | 4 | 3 |
| | Suprachoroidal haemorrhage: involving macula | 8 | 2 |
| | Macular oedema | 4 | 1.5 |
| | Macular hole formation | 6 | 2 |
| | Persistent localised subretinal fluid: peripheral, non-progressive | 3 | 1 |
| | Persistent localised subretinal fluid: submacular | 5 | 2.5 |
| | Retinal re-detachment due to new or worsening PVR | 7 | 1 |
| | Endophthalmitis | 9 | 1 |
| | Sympathetic ophthalmia | 9 | 2 |
| PPV IO | Unintended enlargement of sclerotomy | 2 | 0.5 |
| | Suprachoroidal / subretinal infusion | 4 | 2 |
| | Leaking ports at the end of surgery requiring suturing | 2 | 1 |
| | Cataract development intra-operatively (due to lens touch): Without capsular breach | 4 | 3 |
| | Cataract development intra-operatively (due to lens touch): With capsular breach | 5 | 3 |
| | Intra-operative displacement of perfluorocarbon liquid (PFCL) under the retina | 6 | 3 |

© 2021 Xu ZY et al. *JAMA Ophthalmology*.

| | | | |
|---|---|---|---|
| | Small bubble formation when inserting PFCL | 2 | 2 |
| | 'Sticky' silicone oil at the time of removal | 3 | 3 |
| | Iatrogenic retinal tears | 4 | 1 |
| | Vitreo-retinal incarceration in sclerotomy | 5 | 3 |
| PPV PO | Anterior displacement of tamponade agent: PFCL | 3 | 1 |
| | Anterior displacement of tamponade agent: Silicone oil | 4 | 2 |
| | Subfoveal PFCL | 7 | 2 |
| | Subretinal displacement of silicone oil and heavy silicone oils | 7 | 2.5 |
| | Incomplete removal of tamponade agent | 5 | 3 |
| | Unexplained visual acuity loss associated with insertion / removal of silicone oil | 7 | 2 |
| | Emulsification of tamponade agents | 4 | 2 |
| | Leaky sclerotomy requiring suturing | 3 | 3 |
| | Maculopathy related to light toxicity | 7 | 2 |
| | Maculopathy related to dye toxicity | 7 | 2 |
| | Macular folds: not involving fixation | 4 | 1 |
| | Macular folds: involving fixation | 7 | 2 |
| | Retinal slippage | 6 | 2 |
| | Retinal re-detachment due to new tear formation | 7 | 2.5 |
| | Non-infectious uveitis | 4 | 3 |
| SB IO | Vitreo-retinal incarceration in sclerotomy at time of draining of subretinal fluid | 6 | 2 |
| | Inadvertent scleral perforation when suturing explant | 4 | 3 |
| | Inadvertent drainage of subretinal fluid | 3 | 1 |
| | Air / gas injection behind the lens | 3 | 3 |
| | Sub-retinal injection of air / gas | 6 | 2 |
| | Cataract development intra-operatively (due to lens touch e.g. at time of injection of air/gas): Without capsular breach | 4 | 2 |
| | Cataract development intra-operatively: With capsular breach | 7 | 3 |
| SB PO | Exposure of the buckle | 3 | 2 |
| | Infection of the buckle | 5 | 3 |
| | Migration of the buckle | 4 | 2 |
| | Diplopia / strabismus: Corrected with glasses / prisms | 4 | 2.5 |
| | Diplopia / strabismus: Requiring surgery | 6 | 3 |
| PR IO | 'Fish-egg' gas bubble formation | 3 | 2 |
| | Gas injection behind the lens | 3 | 3 |
| | Sub-retinal injection of gas | 6 | 2 |
| | Cataract development intra-operatively (due to lens touch at the time of gas injection): Without capsular breach | 4 | 2.5 |
| | Cataract development intra-operatively: With capsular breach | 7 | 3 |
| PR PO | New retinal tear formation | 4 | 3 |
| | Retinal re-detachment due to new tear formation | 6 | 4 |

© 2021 Xu ZY et al. *JAMA Ophthalmology*.

## eTable 2b: Delphi Round 2 Results: Median and IQR scores for complications by technique: pars-plana vitrectomy (PPV), scleral buckle (SB), pneumatic retinopexy (PR), and whether intra-operative (IO) or post-operative (PO)

| | Complication | Median | IQR |
|---|---|---|---|
| General IO | Subretinal haemorrhage not involving macula, <= 1 quadrant | 3 | 2 |
| | Subretinal haemorrhage not involving macula, > 1 quadrant | 4 | 2 |
| | Subretinal haemorrhage involving macula, <= 3 disc areas | 7 | 2 |
| | Subretinal haemorrhage involving macula, > 3 disc areas | 8 | 2 |
| | Suprachoroidal haemorrhage not involving macula and no kissing | 5 | 2.75 |
| | Suprachoroidal haemorrhage involving macula or kissing | 9 | 2 |
| General PO | Visual field loss not related to retinal detachment but attributable to surgical procedure: Not affecting driving license | 4 | 2 |
| | Visual field loss not related to retinal detachment but attributable to surgical procedure: Affecting driving license | 7 | 2 |
| | Early hypotony, self-resolving | 2 | 1 |
| | IOL dislocation | 6 | 1 |
| | Suprachoroidal haemorrhage: not involving macula and no kissing | 5 | 2 |
| | Suprachoroidal haemorrhage: involving macula or kissing | 9 | 1 |
| | Persistent localised subretinal fluid: submacular | 5 | 2 |
| PPV IO | Suprachoroidal infusion | 5 | 2 |
| | Subretinal infusion | 5 | 2.75 |
| | Cataract development intra-operatively (due to lens touch): Without capsular breach | 4 | 2 |
| | Cataract development intra-operatively (due to lens touch): With capsular breach | 5 | 1 |
| | Intra-operative displacement of perfluorocarbon liquid (PFCL) under the retina | 6 | 1 |
| | Vitreo-retinal incarceration in sclerotomy | 5 | 2 |
| PPV PO | Subretinal displacement of silicone oil and heavy silicone oils | 7 | 1 |
| | Incomplete removal of tamponade agent | 5 | 1 |
| | Leaky sclerotomy requiring suturing | 3 | 1 |
| | Retinal re-detachment due to new tear formation | 7 | 1 |
| | Non-infectious uveitis | 4 | 1 |
| SB IO | Inadvertent scleral perforation when suturing explant | 4 | 1 |
| | Air / gas injection behind the lens (pre-anterior hyaloid) | 4 | 1 |
| | Cataract development intra-operatively: With capsular breach | 7 | 1 |
| SB PO | Early exposure of the buckle (<1 week) | 5 | 2 |
| | Delayed exposure of the buckle (after 1 week) | 4 | 2 |
| | Infection of the buckle | 5 | 1.75 |
| | Early migration of the buckle (<1 week) | 5 | 2.75 |
| | Delayed migration of the buckle (after 1 week) | 5 | 1 |
| | Diplopia / strabismus: Corrected with glasses / prisms | 4 | 1 |
| | Diplopia / strabismus: Requiring surgery | 6 | 1.75 |
| PR IO | Fish-egg' gas bubble formation not affecting view of retina | 2 | 1 |
| | Fish-egg' gas bubble formation affecting view of retina | 4 | 1 |

© 2021 Xu ZY et al. *JAMA Ophthalmology*.

| | | | |
|---|---|---|---|
| | Gas injection behind the lens (pre-anterior hyaloid) | 4 | 1 |
| | Cataract development intra-operatively (due to lens touch at the time of gas injection): Without capsular breach | 4 | 1 |
| | Cataract development intra-operatively: With capsular breach | 7 | 1 |
| PR PO | New retinal tear formation | 4 | 1 |
| | Retinal re-detachment due to new tear formation | 6 | 2 |

© 2021 Xu ZY et al. *JAMA Ophthalmology*.

**eTable 3: Complications ranked by severity scores and classified as mild (median scores 1-4), moderate (median scores 5-7) and severe (median scores 8-10)**

| Complication | Severity Score |
|---|---|
| ***MILD*** | |
| Chemosis | 1 |
| Subconjunctival haemorrhage | 1 |
| Refractive changes: < 2D | 2 |
| Early hypotony, self-resolving | 2 |
| Early raised IOP, self-resolving | 2 |
| Leaking ports at the end of PPV requiring suturing | 2 |
| Small bubble formation when inserting PFCL in PPV | 2 |
| Unintended enlargement of the sclerotomy in PPV | 2 |
| Fish-egg' gas bubble formation not affecting view of retina in PR | 2 |
| Subretinal haemorrhage not involving macula, ≤ 1 quadrant | 3 |
| Serous choroidal detachment: peripheral | 3 |
| Persistent localised subretinal fluid: peripheral, non-progressive | 3 |
| Anterior displacement of PFCL during PPV | 3 |
| Leaky sclerotomy during PPV requiring suturing | 3 |
| Subretinal haemorrhage not involving macula, > 1 quadrant | 4 |
| Visual field loss attributable to the surgical procedure: Not affecting driving license | 4 |
| Refractive changes: ≥ 2D | 4 |
| Persistently raised IOP manageable with drops | 4 |
| IOL displacement | 4 |
| Macular oedema | 4 |
| Cataract development intra-operatively (due to lens touch): Without capsular breach | 4 |
| Iatrogenic retinal tears following PPV | 4 |
| Anterior displacement of Silicone oil following PPV | 4 |
| Emulsification of tamponade agent following PPV | 4 |
| Macular folds: not involving fixation following PPV | 4 |
| Non-infectious uveitis following PPV | 4 |
| Inadvertent scleral perforation when suturing explant during SB | 4 |
| Air / gas injection behind the lens (pre-anterior hyaloid) during SB or PN | 4 |
| Delayed exposure of the buckle (after 1 week) | 4 |
| Diplopia / strabismus: Corrected with glasses / prisms following SB | 4 |
| Fish-egg' gas bubble formation affecting the view of the retina in PR | 4 |
| New retinal tear formation postoperatively following PR | 4 |
| ***MODERATE*** | |
| Suprachoroidal haemorrhage not involving macula and no kissing* | 5 |
| Persistent localised subretinal fluid: submacular | 5 |
| Cataract development intra-operatively (due to lens touch): With capsular breach during PPV | 5 |
| Suprachoroidal infusion during PPV | 5 |
| Subretinal infusion* during PPV | 5 |
| Vitreo-retinal incarceration in sclerotomy during PPV | 5 |
| Incomplete removal of tamponade agent following PPV | 5 |
| Early exposure of SB (<1 week) | 5 |
| Infection of SB | 5 |
| Early migration of SB(<1 week)* | 5 |
| Delayed migration of SB (after 1 week) | 5 |
| Loss of visual acuity attributable to surgical procedure: Moderate (3-5 lines ETDRS chart) | 6 |
| Persistent hypotony (IOP < 5mmHg) without macular folds | 6 |
| IOL dislocation | 6 |

© 2021 Xu ZY et al. *JAMA Ophthalmology*.

| | |
|---|---|
| ***MODERATE*** | |
| Macular hole formation | 6 |
| Intra-operative displacement of PFCL under the retina during PPV | 6 |
| Retinal slippage during PPV | 6 |
| Vitreo-retinal incarceration in sclerotomy at time of draining of subretinal fluid during SB | 6 |
| Sub-retinal injection of air / gas during SB or PR | 6 |
| Diplopia / strabismus: Requiring surgery post SB | 6 |
| Retinal re-detachment due to new tear formation post PR | 6 |
| Subretinal haemorrhage involving macula, ≤ 3 disc areas | 7 |
| Visual field loss not related to retinal detachment but attributable to surgical procedure: Affecting driving license | 7 |
| Corneal decompensation / severe corneal oedema | 7 |
| Persistently raised IOP requiring surgery | 7 |
| Serous choroidal detachment: large, kissing | 7 |
| Retinal re-detachment due to new or worsening PVR | 7 |
| Subfoveal PFCL post PPV | 7 |
| Subretinal displacement of silicone oil and heavy silicone oils post PPV | 7 |
| Unexplained visual acuity loss associated with insertion / removal of silicone oil post PPV | 7 |
| Maculopathy related to light toxicity post PPV | 7 |
| Maculopathy related to dye toxicity post PPV | 7 |
| Macular folds: involving fixation post PPV | 7 |
| Retinal re-detachment due to new tear formation post PPV | 7 |
| Cataract development intra-operatively: With capsular breach post SB or PR | 7 |
| ***SEVERE*** | |
| Subretinal haemorrhage involving macula, > 3 disc areas | 8 |
| Loss of visual acuity attributable to surgical procedure: Severe (≥ 6 lines on ETDRS chart) | 8 |
| Persistent hypotony with macular folds | 8 |
| Suprachoroidal haemorrhage involving macula or kissing | 9 |
| Suprachoroidal haemorrhage: involving macula or kissing | 9 |
| Endophthalmitis | 9 |
| Sympathetic ophthalmia | 9 |
| Phthisis | 10 |

PPV = pars plana vitrectomy; SB = scleral buckle; PR = pneumatic retinopexy; PFCL = perfluorocarbone liquid.
*Interquartile range = 2.75 (i.e. did not reach consensus)

© 2021 Xu ZY et al. *JAMA Ophthalmology*.
